# Nanotechnologies in Cancer

**DOI:** 10.1155/2013/604293

**Published:** 2013-05-13

**Authors:** Giuseppe De Rosa, Michele Caraglia, Stefano Salmaso, Tamer Elbayoumi

**Affiliations:** ^1^Department of Pharmacy, University Federico II of Naples, Via Montesano 49, 80131 Naples, Italy; ^2^Department of Biochemistry, Biophysics and General Pathology, Second University of Naples, Via S. M. Costantinopoli 16, 80138 Naples, Italy; ^3^Department of Pharmaceutical and Pharmacological Sciences, University of Padova, Via F. Marzolo 5, 35131 Padova, Italy; ^4^Department of Pharmaceutical Sciences, Midwestern University, 19555 North 59th Avenue, Glendale, AZ 85308, USA

Cancer is today the major cause of morbidity and mortality in western and industrialized countries. The use of drugs for the therapeutic treatment of cancer raises important issues about their toxicity on normal cells and, more in general, on their systemic side effects.

Issues about systemic toxicities have been faced with both first generation anticancer drugs and with more recent drugs that operate through specific targets, with the latter maintaining the homeostasis of several normal tissues. The emergence of the nanomedicine has opened a novel scenario in the use of all anticancer agents with the possibility to improve their efficacy and to reduce their side effects due to their distribution in normal tissues. Products based on nanotechnological carriers have entered the clinical practice, and a huge number of studies have been performed in order to optimize the application of nanomedicines in cancer treatment. Although these nanotechnology-based systems are still far to fully comply the idea of the “magic bullet,” the advantages offered by this approach are clearly promising.

This special issue covers different aspects related to the exploitation of nanotechnology-based systems for cancer treatment, including the design and features of multifunctional nanocarriers, the drug targeting concept, the gene therapy, the toxicity of nanomaterials, and the more recent clinical studies that have determined a glimmer of hope for cancer patients.

Liposomes are among the first nanotechnological-based platforms ever developed for cancer therapy. One of the major limitations in the clinical use of liposomes and other nanoparticles is their short plasma half-life due to the rapid opsonization process that yields their removal from bloodstream and degradation by macrophages from reticular endothelial system. On the basis of these considerations, “stealth” nanocarriers have been promptly developed through conjugation of hydrophilic polymers, such as polyethylene glycol (PEG), on the particle surface. The review of S. Salmaso and P. Caliceti describes the basic concept underlining the “stealth” properties of drug nanocarriers, the parameters influencing the polymer coating performance in terms of opsonins/macrophages interaction with the colloid surface, the most commonly used materials for the coating process, and the outcomes of this peculiar procedure.

One of the first “stealth” nanocarriers loaded with anticancer drug that has achieved the clinical practice was the pegylated liposomal doxorubicin (PLD). The paper by C. Pisano et al. describes the role and clinical indications of PLD in ovarian cancer. PLD was firstly approved for platinum-refractory ovarian cancer and then received full approval for platinum-sensitive recurrent disease. Recently, it was demonstrated that the combination of PLD with platinum has similar activity but less toxicity than the combination containing free doxorubicin triggering new interest on PLD also in the first line of treatment of this tumour. Another clinical indication of PLD is the treatment of metastatic or locally advanced breast cancer when the maximal allowed cumulative doses of doxorubicin administered to patients is reached. The paper by J. Lao et al. summarizes the main results achieved with the use of PLD in this setting of patients underlining the loss of cardiotoxicity with the preservation of clinical activity if compared to free doxorubicin. Moreover, interesting results have been recorded by combining anti-HER-2 antibodies (trastuzumab) with PLD in the treatment of both locally advanced and metastatic breast cancer enlightening the potential advantages of the combination of these drugs (both cardiotoxic) in these two clinical settings.

An important concern that limits the therapeutic profile of doxorubicin and other anticancer agents is the development of innate or acquired tumour resistance that is mediated by several mechanisms. The paper by D. Ayers and A. Nasti describes the different mechanisms by which tumour cells generate the resistance to anticancer agents and the strategies to overcome the refractoriness of cancer cells. In details, the authors discuss the limits and advantages of different nanotechnological devices used to deliver cytotoxic drugs or nucleic acids (such as micro-RNAs or siRNAs) that target specific molecular resistance factors.

Another important limitation to the effective therapeutic activity of anticancer drugs is the inability of some molecules to overcome anatomic barriers, such as the blood-brain barrier, and to accumulate in the subarachnoidal or leptomeningeal spaces that can be sites of dissemination of brain or extra-brain tumours. The paper by A. Silvani et al. describes the role of liposomal arabinoside cytosine (AraC) in the treatment of neoplastic meningosis including an unpublished prospective trial performed in the Italian region Lombardia and a short review of the data reported by other already published clinical studies.

The paper from I. Cucinotto et al. reports and discusses the most recent findings on the clinical use of nanoparticle albumin-bound paclitaxel (nab-paclitaxel), also known with the commercial name of Abraxane. This drug is at the moment approved for the treatment of metastatic breast cancer and nonsmall cell lung cancer. However, this nanotechnology-based drug is very promising also for the treatment of other human neoplasms, such as pancreatic cancer or metastatic melanoma, which generally are considered refractory to treatment with conventional anticancer agents. In this view, the paper of J. R. Viola et al. provides a short introduction to the mechanisms of melanomagenesis, discussing the shortcomings of current therapeutic approaches ascribed to the existence of a wide range of mutations associated with this cancer. Authors highlight alternative approaches for treatment of melanomas based on the use of therapeutically active nucleic acids. The delivery of nucleic acid nanopharmaceutics is brought into perspective as a novel highly selective antimelanoma therapeutic approach whilst avoiding unwanted and toxic side effects. The possibilities for melanoma selective targeting are discussed together with latest reports of advanced clinical applications.

Also target-based agents need to be specifically delivered to tumour tissues and in this regard G. De Rosa et al. provide a comprehensive article on the clinical applications of bisphosphonates (BPs) starting from their use as inhibitors of bone resorption up to their novel therapeutic indications as anticancer drugs. In detail, nitrogen-containing BPs (N-BPs) induce apoptosis in a variety of cancer cells in vitro and in preclinical settings and show a very intriguing antiangiogenic activity. Unfortunately, clinical anticancer activity of N-BPs is far to be demonstrated. In this light, the authors describe how nanotechnology can provide carriers to limit BP accumulation into the bone, thus increasing drug level in extra-skeletal sites of the body to directly kill cancer cells. On the other hand, BPs can also be used as targeting agents to specifically deliver nanocarriers loaded with anticancer drugs in the bone tissue for the treatment of bone tumours or metastases.

The active targeting of nanoparticles is an effective strategy to increase the uptake of anti-cancer drug-loaded vehicles by tumour cells. It is based on the decoration of nanoparticles with specific ligands such as peptides or antibodies raised against tumour-associated antigens (molecules with higher expression on tumour cells than in normal counterparts).

In this light, S. Arpicco et al. review the use of hyaluronic acid (HA) as a unique targeting agent for the recognition of cancer cells due to the high expression levels of its receptor (named CD44) on tumour cell surface. The CD44 receptor is found at low levels on the surface of epithelial, haematopoietic, and neuronal cells, but it is overexpressed in many cancer cells and on cancer stem cells. This review describes the approaches used for the preparation and investigation of lipid-based nanovectors decorated with HA for the active delivery of a variety of therapeutic molecules in the treatment of human cancer.

Other strategies in the development of nanotechnological devices include the multifunctional decoration with different moieties that allow both the detection and the treatment of cancer cells (theranostic devices). In this view, the paper by F. Perche and V. P. Torchilin describes multifunctional liposomal nanocarriers that combines long blood circulation and selective accumulation to the tumor lesions based upon remote-controlled or tumour stimuli-sensitive extravasation from blood to the tumour tissue and internalization motifs to move from tumour bounds and/or tumor intercellular space to the cytoplasm of cancer cells.

Finally, nanovectors are not completely inert materials and can be endowed with intrinsic cytotoxicity that causes, sometimes, potential deleterious effects in normal tissues. In this light, D. De Stefano et al. describe the main mechanisms by which nanosized materials can induce cell death, such as apoptosis, mitotic catastrophe, authophagy, necrosis, and pyroptosis. The understanding of these mechanisms is mandatory for a safe use of nanocarriers. The authors describe all the variables that can affect nanocarrier cytotoxicity underlining the need for generally accepted guidelines for the development and use of nanotechnological devices.

We believe that this special issue can be of great interest for the readers in depicting the most recent advances generated by basic, translational, and clinical research focused on the development and use of nanocarriers for the delivery of anticancer agents. The special issue thoroughly reports the outcomes derived from basic and preclinical studies and the main limitations emerged from both clinical trials and practice. The criticisms derived from the clinics need to be regarded as crucial starting points for the optimization of the nanotechnological drug delivery systems. In other words, bidirectional flow of information from the bench to the bedside and back again to the bench is pivotal to offer improved nanomedicine-based strategies of treatment of cancer patients.

*Giuseppe De Rosa* *Giuseppe De Rosa* *Michele Caraglia* *Michele Caraglia* *Stefano Salmaso* *Stefano Salmaso* *Tamer Elbayoumi* *Tamer Elbayoumi*

